# 1,5-Bis(2,5-dimethyl-1*H*-pyrrol-1-yl)naphthalene

**DOI:** 10.1107/S1600536809038999

**Published:** 2009-10-03

**Authors:** A. C. Santos, M. Ramos Silva, P. V. Monsanto, A. Matos Beja, A. J. F. N. Sobral

**Affiliations:** a Departamento de Qυ’imica, Faculdade de Ciências e Tecnologia, Universidade de Coimbra, P-3004-535 Coimbra, Portugal; b CEMDRX, Departamento de Física, Faculdade de Ciências e Tecnologia, Universidade de Coimbra, P-3004-516 Coimbra, Portugal; c Forensic Toxicology Service, National Institute of Legal Medicine, Center Branch, P-3000-213 Coimbra, Portugal

## Abstract

In the title compound, C_22_H_22_N_2_, the asymmetric unit contains one half-mol­ecule. A crystallographic inversion centre is located at the mid-point of the bond common to both rings, in the central naphthalene unit. Quantum-mechanical *ab initio* calculations on the isolated mol­ecule showed that the minimum energy configuration occurs when the naphthalene ring system and the pyrrolyl groups deviate only slightly from perpendicularity. In the crystal, due to the effects of crystal packing, the mol­ecule deviates by approximately 4° from the *a priori* expected ideal value of 90° [C—C—N—C torsion angle = 86.11 (15)°].

## Related literature

For related compounds, see: Andrade *et al.* (2008 ▶); Ramos Silva *et al.* (2002 ▶); Sobral (2006 ▶); Sobral & Rocha Gonsalves (2001*a* ▶,*b* ▶). For the *ab initio* calculation method, see: Schmidt *et al.* (1993 ▶).
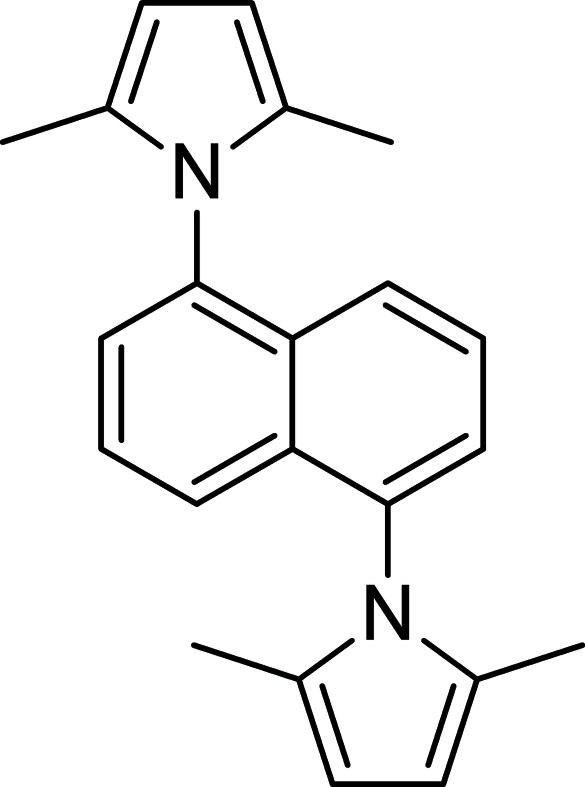


## Experimental

### 

#### Crystal data

C_22_H_22_N_2_
*M* *_r_* = 314.42
Monoclinic, 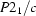

*a* = 8.7562 (3) Å
*b* = 7.2806 (2) Å
*c* = 14.1380 (5) Å
β = 101.4721 (16)°
*V* = 883.30 (5) Å^3^
*Z* = 2
Mo *K*α radiation
μ = 0.07 mm^−1^
*T* = 293 K
0.30 × 0.30 × 0.02 mm

#### Data collection

Bruker APEXII CCD area-detector diffractometer
Absorption correction: multi-scan (*SADABS*; Sheldrick, 2000 ▶) *T*_min_ = 0.892, *T*_max_ = 0.999
23689 measured reflections
2415 independent reflections
1798 reflections with *I* > 2σ(*I*)
*R*_int_ = 0.028

#### Refinement

*R*[*F* ^2^ > 2σ(*F* ^2^)] = 0.044
*wR*(*F* ^2^) = 0.160
*S* = 1.11
2415 reflections
111 parameters
H-atom parameters constrained
Δρ_max_ = 0.19 e Å^−3^
Δρ_min_ = −0.20 e Å^−3^

### 

Data collection: *APEX2* (Bruker, 2003 ▶); cell refinement: *SAINT* (Bruker, 2003 ▶); data reduction: *SAINT*; program(s) used to solve structure: *SHELXS97* (Sheldrick, 2008 ▶); program(s) used to refine structure: *SHELXL97* (Sheldrick, 2008 ▶); molecular graphics: *PLATON* (Spek, 2009 ▶); software used to prepare material for publication: *SHELXL97*.

## Supplementary Material

Crystal structure: contains datablocks global, I. DOI: 10.1107/S1600536809038999/pk2188sup1.cif

Structure factors: contains datablocks I. DOI: 10.1107/S1600536809038999/pk2188Isup2.hkl

Additional supplementary materials:  crystallographic information; 3D view; checkCIF report

## Figures and Tables

**Fig. 1.**
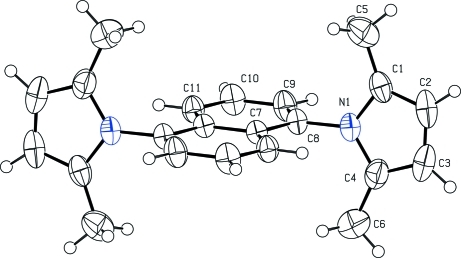
Ellipsoid plot of the title compound. Displacement ellipsoids are drawn at the 50% level. Unlabelled atoms are generated by inversion through the origin.

## supplementary crystallographic information

### Comment

Complex pyrroles are important synthons in macromolecular chemistry, environmental chemistry, medical chemistry and nano-technologies based on polymeric organic materials. Following our endeavor in synthesizing new pyrrolic compounds for material chemistry (Andrade *et al.*, 2008; Ramos Silva *et al.*, 2002; Sobral & Rocha Gonsalves 2001*a*, 2001*b*; Sobral, 2006), we prepared the title compound, by the Paal-Knorr methodology, using iodine as catalyst. Each molecule contains a crystallographic inversion centre at the mid-point of the bond common to both rings of the naphthalene moiety.  All bond lengths and valency angles of the molecule lie within the expected range of values for naphtalene derivatives.

Approximate free rotation of the pyrrolyl group around the formal σ C—N bond is expected. Thus, the conformation observed for such groups in the solid state should be determined by steric rather than electronic effects. We observe in this structure a value of 86.11 (15)° for the C9–C8–N1–C1 dihedral angle, which is close to the a priori expected ideal value of 90° where the steric effects should be at a minimum.

In order to gain some insight into how the crystal packing might affect the molecular geometry we have performed a quantum chemical calculation on the equilibrium geometry of the free molecule. These calculations were performed with the computer program GAMESS (Schmidt *et al.*, 1993). A molecular orbital Roothan Hartree-Fock method was used with an extended 6–31 G(d,p) basis set. Tight conditions for convergence of both the self-consistent field cycles and maximum density and energy gradient variations were imposed (10^-6^atomic units). The program was run on the Milipeia cluster of UC-LCA (using 16 Opteron cores, 2.2 GHz runing Linux).

The *ab*-*initio* calculations reproduce well the observed experimental bond length and angles of the molecule. All angles match the experimental values within 1°. Calculated and experimental bond distances agree within 0.023 Å.  The calculated C9–C8–N1–C1 dihedral angle is 91.82°, a value closer to the ideal value of 90° than the experimental value in the solid state.

A check for weak intermolecular interactions in the crystal on the basis of short contacts revealed that a possible C—H···π interaction may exist between atoms C2 and the pyrrole ring [C2—H2···C_g_: 3.7791 (16) Å, 159°]

### Experimental

0.680 g (4.3 mmol) of 1,4-phenylenedimethanamine and 1 ml (8.5 mmol) of hexane-2,5-dione were dissolved in 20 ml of tetrahydrofuran, under nitrogen atmosphere. 0.172 g (0.678 mmol) of iodine was added to the stirred solution at 40°C. The procedure was monitored by TLC. After completion of the reaction (6 h), 20 ml of dichlorometane were added to the mixture. The resulting mixture was washed successively with 5% Na_2_S_2_O_3_ solution (2 ml), NaHCO_3_ solution (2 ml) and brine (2 ml). The organic layer was then dried with anhydrous sodium sulfate and concentrated. The product was purified by flash chromatography in silica gel 60H FLUKA/dichloromethane and recrystallized in cold dichloromethane, by slow solvent evaporation, to give needle shape crystals 0.473 grams corresponding to 1.5 mmoles (%) = 35; GC/MS (100 µmol/ml in CH_2_Cl_2_) m/*z* = 314; ^1^H-NMR (0.1 *M* in CDCl_3_, 499.428 MHz) σ 1.96 (s, 12H, Methyl), σ 5.34 (s, 4H, pyrrole), σ 7.25 (dd, 2H, Aromatic, J = 0.99, J = 7.49 Hz), 7.48 (dd, 2H, Aromatic, J = 0.99 Hz, J = 6.99 Hz), 7.55 (t, 2H, Aromatic, J = 7.0 Hz); ^13^C - NMR (0.1 *M* in CDCl3, 125.692 MHz) σ 12.5 (Methyl), σ 105.6 (Pyrrole), σ 129.8 (Pyrrole), σ 126.7 (Aromatic), σ 132.7 (Aromatic). Melting point: Decomposes at 288 °C.

### Refinement

The methyl H atoms were constrained to an ideal geometry (C—H = 0.96 Å) with *U*_iso_(H)= 1.5*U*_eq_(C), but were allowed to rotate freely about the C—C bonds. All remaining H atoms were placed in geometrically idealized positions and constrained to ride on their parent atoms with *U*_iso_(H) = 1.2*U*_eq_(parent atom).

### Crystal data

**Table:**

C_22_H_22_N_2_	*F*(000) = 336
*M**_r_* = 314.42	*D*_x_ = 1.182 Mg m^−^^3^
Monoclinic, *P*2_1_/*c*	Mo *K*α radiation, λ = 0.71073 Å
Hall symbol: -P 2ybc	Cell parameters from 8233 reflections
*a* = 8.7562 (3) Å	θ = 2.4–28.6°
*b* = 7.2806 (2) Å	µ = 0.07 mm^−^^1^
*c* = 14.1380 (5) Å	*T* = 293 K
β = 101.4721 (16)°	Plate, brown
*V* = 883.30 (5) Å^3^	0.30 × 0.30 × 0.02 mm
*Z* = 2	

### Data collection

**Table:**

Bruker APEXII CCD area-detector diffractometer	2415 independent reflections
Radiation source: fine-focus sealed tube	1798 reflections with *I* > 2σ(*I*)
graphite	*R*_int_ = 0.028
φ and ω scans	θ_max_ = 29.5°, θ_min_ = 2.4°
Absorption correction: multi-scan (*SADABS*; Sheldrick, 2000)	*h* = −12→11
*T*_min_ = 0.892, *T*_max_ = 0.999	*k* = −10→9
23689 measured reflections	*l* = −18→19

### Refinement

**Table:**

Refinement on *F*^2^	Primary atom site location: structure-invariant direct methods
Least-squares matrix: full	Secondary atom site location: difference Fourier map
*R*[*F*^2^ > 2σ(*F*^2^)] = 0.044	Hydrogen site location: inferred from neighbouring sites
*wR*(*F*^2^) = 0.160	H-atom parameters constrained
*S* = 1.11	*w* = 1/[σ^2^(*F*_o_^2^) + (0.0861*P*)^2^ + 0.0939*P*] where *P* = (*F*_o_^2^ + 2*F*_c_^2^)/3
2415 reflections	(Δ/σ)_max_ < 0.001
111 parameters	Δρ_max_ = 0.19 e Å^−^^3^
0 restraints	Δρ_min_ = −0.20 e Å^−^^3^

### Special details

**Table:**

Geometry. All e.s.d.'s (except the e.s.d. in the dihedral angle between two l.s. planes) are estimated using the full covariance matrix. The cell e.s.d.'s are taken into account individually in the estimation of e.s.d.'s in distances, angles and torsion angles; correlations between e.s.d.'s in cell parameters are only used when they are defined by crystal symmetry. An approximate (isotropic) treatment of cell e.s.d.'s is used for estimating e.s.d.'s involving l.s. planes.
Refinement. Refinement of *F*^2^ against ALL reflections. The weighted *R*-factor *wR* and goodness of fit *S* are based on *F*^2^, conventional *R*-factors *R* are based on *F*, with *F* set to zero for negative *F*^2^. The threshold expression of *F*^2^ > σ(*F*^2^) is used only for calculating *R*-factors(gt) *etc*. and is not relevant to the choice of reflections for refinement. *R*-factors based on *F*^2^ are statistically about twice as large as those based on *F*, and *R*- factors based on ALL data will be even larger.

### Fractional atomic coordinates and isotropic or equivalent isotropic displacement parameters (Å^2^)

**Table:**

	*x*	*y*	*z*	*U*_iso_*/*U*_eq_	
N1	−0.25783 (11)	0.16842 (14)	−0.15743 (7)	0.0413 (3)	
C1	−0.37264 (14)	0.28237 (17)	−0.13576 (10)	0.0457 (3)	
C2	−0.40388 (17)	0.41020 (19)	−0.20743 (11)	0.0581 (4)	
H2	−0.4765	0.5046	−0.2117	0.070*	
C4	−0.21687 (17)	0.2256 (2)	−0.24234 (10)	0.0543 (4)	
C3	−0.3073 (2)	0.3747 (2)	−0.27363 (11)	0.0644 (5)	
H3	−0.3054	0.4416	−0.3294	0.077*	
C5	−0.4403 (2)	0.2558 (2)	−0.04885 (14)	0.0677 (5)	
H5A	−0.5235	0.3419	−0.0496	0.102*	
H5B	−0.3612	0.2752	0.0079	0.102*	
H5C	−0.4800	0.1329	−0.0484	0.102*	
C6	−0.0984 (3)	0.1270 (3)	−0.28455 (14)	0.0867 (6)	
H6A	−0.0887	0.1846	−0.3441	0.130*	
H6B	−0.1298	0.0015	−0.2966	0.130*	
H6C	0.0001	0.1310	−0.2403	0.130*	
C7	−0.03746 (12)	0.07020 (14)	−0.03141 (8)	0.0338 (3)	
C8	−0.18058 (13)	0.02723 (15)	−0.09515 (8)	0.0374 (3)	
C9	−0.24388 (15)	−0.14418 (17)	−0.09648 (10)	0.0472 (3)	
H9	−0.3369	−0.1703	−0.1389	0.057*	
C10	−0.16905 (15)	−0.28164 (17)	−0.03401 (10)	0.0478 (3)	
H10	−0.2135	−0.3978	−0.0352	0.057*	
C11	−0.03195 (14)	−0.24638 (15)	0.02834 (9)	0.0403 (3)	
H11	0.0162	−0.3387	0.0691	0.048*	

### Atomic displacement parameters (Å^2^)

**Table:**

	*U*^11^	*U*^22^	*U*^33^	*U*^12^	*U*^13^	*U*^23^
N1	0.0373 (5)	0.0397 (5)	0.0418 (6)	0.0034 (4)	−0.0041 (4)	0.0032 (4)
C1	0.0380 (6)	0.0377 (6)	0.0544 (7)	0.0016 (5)	−0.0074 (5)	−0.0072 (5)
C2	0.0550 (9)	0.0415 (7)	0.0643 (9)	0.0071 (6)	−0.0209 (7)	−0.0012 (6)
C4	0.0542 (8)	0.0616 (9)	0.0425 (7)	0.0017 (6)	−0.0016 (6)	0.0083 (6)
C3	0.0725 (10)	0.0591 (9)	0.0505 (8)	−0.0008 (7)	−0.0144 (7)	0.0161 (7)
C5	0.0623 (10)	0.0612 (9)	0.0825 (11)	0.0101 (7)	0.0214 (8)	−0.0053 (8)
C6	0.0921 (14)	0.1113 (16)	0.0625 (10)	0.0227 (12)	0.0292 (10)	0.0146 (10)
C7	0.0322 (6)	0.0315 (5)	0.0362 (6)	0.0009 (4)	0.0033 (4)	0.0000 (4)
C8	0.0344 (6)	0.0360 (6)	0.0390 (6)	0.0030 (4)	0.0003 (5)	0.0012 (4)
C9	0.0376 (7)	0.0421 (6)	0.0551 (7)	−0.0048 (5)	−0.0071 (5)	−0.0012 (5)
C10	0.0444 (7)	0.0332 (6)	0.0611 (8)	−0.0075 (5)	−0.0012 (6)	0.0011 (5)
C11	0.0401 (6)	0.0316 (5)	0.0468 (7)	0.0005 (4)	0.0026 (5)	0.0042 (4)

### Geometric parameters (Å, °)

**Table:**

N1—C4	1.3835 (17)	C6—H6A	0.9600
N1—C1	1.3840 (16)	C6—H6B	0.9600
N1—C8	1.4326 (14)	C6—H6C	0.9600
C1—C2	1.3628 (19)	C7—C11^i^	1.4164 (15)
C1—C5	1.479 (2)	C7—C8	1.4253 (15)
C2—C3	1.404 (3)	C7—C7^i^	1.426 (2)
C2—H2	0.9300	C8—C9	1.3642 (17)
C4—C3	1.365 (2)	C9—C10	1.4075 (18)
C4—C6	1.481 (2)	C9—H9	0.9300
C3—H3	0.9300	C10—C11	1.3649 (17)
C5—H5A	0.9600	C10—H10	0.9300
C5—H5B	0.9600	C11—C7^i^	1.4164 (15)
C5—H5C	0.9600	C11—H11	0.9300
			
C4—N1—C1	109.68 (11)	C4—C6—H6A	109.5
C4—N1—C8	125.23 (11)	C4—C6—H6B	109.5
C1—N1—C8	124.69 (11)	H6A—C6—H6B	109.5
C2—C1—N1	106.98 (13)	C4—C6—H6C	109.5
C2—C1—C5	130.75 (14)	H6A—C6—H6C	109.5
N1—C1—C5	122.26 (12)	H6B—C6—H6C	109.5
C1—C2—C3	108.15 (13)	C11^i^—C7—C8	122.42 (10)
C1—C2—H2	125.9	C11^i^—C7—C7^i^	119.26 (12)
C3—C2—H2	125.9	C8—C7—C7^i^	118.32 (12)
C3—C4—N1	106.65 (14)	C9—C8—C7	120.88 (10)
C3—C4—C6	131.51 (15)	C9—C8—N1	120.47 (10)
N1—C4—C6	121.82 (13)	C7—C8—N1	118.64 (10)
C4—C3—C2	108.53 (13)	C8—C9—C10	120.31 (11)
C4—C3—H3	125.7	C8—C9—H9	119.8
C2—C3—H3	125.7	C10—C9—H9	119.8
C1—C5—H5A	109.5	C11—C10—C9	120.76 (11)
C1—C5—H5B	109.5	C11—C10—H10	119.6
H5A—C5—H5B	109.5	C9—C10—H10	119.6
C1—C5—H5C	109.5	C10—C11—C7^i^	120.47 (11)
H5A—C5—H5C	109.5	C10—C11—H11	119.8
H5B—C5—H5C	109.5	C7^i^—C11—H11	119.8
			
C4—N1—C1—C2	0.28 (14)	C11^i^—C7—C8—C9	179.75 (12)
C8—N1—C1—C2	173.32 (11)	C7^i^—C7—C8—C9	−0.4 (2)
C4—N1—C1—C5	−179.96 (13)	C11^i^—C7—C8—N1	−0.83 (18)
C8—N1—C1—C5	−6.91 (19)	C7^i^—C7—C8—N1	179.03 (12)
N1—C1—C2—C3	−0.09 (15)	C4—N1—C8—C9	−101.92 (16)
C5—C1—C2—C3	−179.82 (15)	C1—N1—C8—C9	86.11 (15)
C1—N1—C4—C3	−0.36 (16)	C4—N1—C8—C7	78.66 (16)
C8—N1—C4—C3	−173.36 (11)	C1—N1—C8—C7	−93.32 (14)
C1—N1—C4—C6	−178.79 (15)	C7—C8—C9—C10	0.5 (2)
C8—N1—C4—C6	8.2 (2)	N1—C8—C9—C10	−178.89 (12)
N1—C4—C3—C2	0.30 (17)	C8—C9—C10—C11	−0.4 (2)
C6—C4—C3—C2	178.52 (17)	C9—C10—C11—C7^i^	0.1 (2)
C1—C2—C3—C4	−0.14 (17)		

Symmetry codes: (i) −*x*, −*y*, −*z*.
